# Annotations

**Published:** 1899-10-14

**Authors:** 


### Annotations.

### The Hospital Reform Conference.

The very interesting paper read by Mr. Douglas Dent at the Hospital Reform Conference, of which a report appears in another column, placed in a very clear light the necessity of proper inquiry being made into the circumstances of all applicants for hospital treatment, always excepting, of course, the case of those who suffer from accident or emergency. So far as his paper went we readily agree with almost every word he said. Dealing in the abstract with the wrong done by indiscriminate relief, with the evil wrought, not only among hospital patients, but among the great mass of the semi-poor, who by the hospitals are tempted into pauperism, and with the

ANNOTATIONS—*continued.*

injury inflicted upon the whole medical profession, both within and without the hospitals, by the present system, he showed plainly that the root of the mischief lies in the free and indiscriminate manner in which the hospitals dispense their charity, and that the very first step in reform depends upon inquiry into the circumstances of the patients. This, surely, is all true. But he went further, and showed that to be effective such inquiry must be thorough, and he treated with telling ridicule much of the pseudo-inquiry which is now current. Again, we cannot but agree with the principles so broadly laid down. Even beyond being thorough, inquiry must, he said, be universal, and must be conducted on the same basis at all institutions, so as to avoid that competition between the hospitals which at present makes the "hospital sneak" the master of the situation. To all these propositions we think that a general acquiescence should be accorded. In fact, a general acquiescence to them did seem to be most heartily accorded by the fifteen or sixteen people who listened to the reading of the paper. One could not but feel, however, that such a mode of discussing the question was somewhat lacking in the practical. As we applauded the sentiment it was easy to feel, as those about us appeared to do, that now at last the problem was solved. But such a paper has, we fear, but little touch with practical realities, and we cannot but feel that when hospital reformers walk into the out-patient waiting-rooms of our great hospitals and endeavour to apply the "almoner" principle to the vast hordes of sick and poor that they will find there, when, in other words, after their airy theories, they touch the ground and come down again to mother earth, they will find the task a heavier one than they seem to think. We wish it were otherwise. The almoner's principle is quite ideal. But there are many things to be considered. There is the urgency of the cases and their enormous multitude; there is the difficulty of definition of the "fit case;" there is the doubt as to what sort of medical attendance is to be considered "adequate" for the case that is refused admission; there is the fact that there is not, and can never be, any distinct dividing line separating the fit from the unfit, so that each case of this great multitude must be considered on its own merits; and there are numberless other questions on each of which radical differences may arise. Mr. Dent said that he preferred to deal with general principles on which all men can unite, and to put on one side the consideration of details in which they were so apt to differ—and he was wise.

**The "Sun" on Colour-blindness.**

Commenting upon an article entitled "Colour-blind Sailors," which appeared in The Hospital, September 30th, the *Sun* writes as follows:—

"No check, says The Hospital, of any kind, seems yet to have been placed upon the entrance of colour-blind or poor-sighted boys to the Navy, so that there are constantly among our seamen a large number of colour-blind and nautically-blind men who may continue in the Service to the end of their days.

"'I have no idea why The Hospital should make such a statement,' said an official at the Admiralty Recruiting Department to a *Sun* representative. 'Our sight and colour tests are more stringent than in any other branch of the Service.'

"'And they are always applied?'

"'Always. We are more particular about that than anything else, and never allow the tests to be shirked. No lad with really defective sight, or who is colour-blind, has any chance of getting into the Royal Navy.'

"Is The Hospital prepared to substantiate its allegations?"

But *we* never alluded to the Royal Navy. *We* did not even use the word navy from one end of the paragraph to the other, never to mention putting it in capitals. *We* did not put a capital to the word "service." All this is the *Sun's* doing, not ours. We specifically mentioned, twice over, that we were writing about the "mercantile marine"; we said that the sailors of whom we spoke have to undergo an examination of their eyesight when they "rise in the ranks and apply for officers' certificates," which, of itself, should have been sufficient to show that we were not speaking of the navy; and, finally, as we more than once pointed out, it was the Board of Trade that we held up to obloquy. Surely the Royal Navy is not under the Board of Trade! We are sorry that the *Sun* should have taken the trouble to send all the way to the Admiralty to inquire about the matter; but we are still more sorry that its representative should, by his carelessness, have so misrepresented us to its readers and to the Admiralty officials.

**A Disjointed Profession.**

As illustrating the curious want of unity which pervades the medical profession, and the difficulty of drawing practitioners together for mutual support, we would refer to certain suggestions which have been put forward with the object of "organising" the profession. Truly this would seem a most laudable object, and, if arranged on a proper basis, one with which all of us ought to sympathise. We are told that, in consequence of the evils from which the general practitioner suffers, and the steady encroachments on his prerogatives which are continually being made, "it is absolutely essential that we stand shoulder to shoulder," and we are further assured that if we do this we shall not only be able to resist aggression, but be in a position to uphold the dignity of the profession. All these are noble sentiments, and we thank the Medical Guild of Manchester for formulating them so clearly. Unfortunately, just when unity and organisation seem within the range of possibility there comes a letter from a "General Practitioner," who, writing in one of our contemporaries, pricks the bubble of our hopes, declaring that this suggestion "is really too funny," and going on to say: "The provident dispensary surgeons in Manchester take a leading part in the council of the Medical Guild. The provident dispensaries have done enormous injury to the general practitioners, yet those connected with them are endeavouring to promote 'cohesion, organisation, and *esprit de corps*' amongst general practitioners. Is one single word more necessary?" Now, who is right? Really, we almost despair of our profession.

